# LncRNA4930473A02Rik promotes cardiac hypertrophy by regulating TCF7 via sponging miR-135a in mice

**DOI:** 10.1038/s41420-021-00775-8

**Published:** 2021-12-07

**Authors:** Jing Ren, Hanping Qi, Chao Song, Lina Ba, Renling Liu, Xiang Feng, Lixin Wang, Meitian Zhang, Yawen Xie, Hongli Sun

**Affiliations:** Department of Pharmacology, Harbin Medical University-Daqing, Daqing, Heilongjiang 163319 China

**Keywords:** Non-coding RNAs, Cardiovascular diseases

## Abstract

Cardiac hypertrophy is a common pathological change accompanied by various cardiovascular diseases; however, its underlying mechanisms remain elusive. Mounting evidence indicates that long non-coding RNAs (lncRNAs) are novel transcripts involved in regulating multiple biological processes. However, little is known about their role in regulating cardiac hypertrophy. This study revealed a novel lncRNA4930473A02Rik (abbreviated as lncRNAA02Rik), which showed considerably increased expression in hypertrophic mouse hearts in vivo and angiotensin-II (Ang-II)-induced hypertrophic cardiomyocytes in vitro. Notably, lncRNAA02Rik knockdown partly ameliorated Ang-II induced hypertrophic cardiomyocytes in vitro and hypertrophic mouse heart function in vivo, whereas lncRNAA02Rik overexpression promoted cardiac hypertrophy in vitro. Furthermore, lncRNAA02Rik acted as a competing endogenous RNA by sponging miR-135a, while forced expression of lncRNAA02Rik could repress its activity and expression. Furthermore, forcing miR-135a overexpression exerted a significant protective effect against cardiac hypertrophy by inhibiting the activity of its downstream target TCF7, a critical member of Wnt signaling, and the protective effect could be reversed by AMO-135a. Luciferase assay showed direct interactions among lncRNAA02Rik, miR-135a, and TCF7. Altogether, our study demonstrated that lncRNAA02Rik upregulation could promote cardiac hypertrophy development via modulating miR-135a expression levels and TCF7 activity. Therefore, lncRNAA02Rik inhibition might be considered as a novel potential therapeutic strategy for cardiac hypertrophy.

## Introduction

The heart initially develops cardiac hypertrophy under excessive stress as an adaptive response to reduce wall stress and prevent cardiac dysfunction [1, 2]. However, sustained overload causes cardiac dysfunction, eventually leading to heart failure and even sudden death [3–5]. Therefore, it is of great importance to explore and uncover the molecular mechanisms of cardiac hypertrophy.

Long non-coding RNAs (lncRNAs) are a group of transcribed RNA molecules with more than 200 nucleotides, which have no obvious protein-coding potential [6]. Previous studies have indicated that the aberrant expression of lncRNAs could be critical in multiple cardiovascular diseases [7]. For instance, Lv et al. reported that lncRNAPlscr4 controlled cardiac hypertrophy by regulating miR-214 [8]. LncRNAs have various pivotal roles with multiple mechanisms, including chromatin remodeling and RNA stability, etc [9–12]. Particularly, lncRNAs are thought to function as a “sponge” to absorb microRNAs (miRNAs) and affect post-transcriptional processing; for example, lncRNAGAS5 acts as a competing endogenous RNA (ceRNA) by sponging miR-222 [13].

MiRNAs, members of small ncRNAs, have been observed to negatively modulate gene expression primarily by base pairing to the 3′-untranslated region (UTR) of target mRNAs, leading to mRNA cleavage and translation repression [14–16]. Functionally speaking, multiple cardiovascular diseases have now been associated with dysregulated miRNA expression [17, 18]. Our previous study indicated that miR-103 negatively affected cardiac hypertrophy via regulating Trpv3 [19]. Yuan et al. have reported that miR-21 contributed to cardiac fibrosis by targeting Smad7 [20]. The Wnt signaling pathway regulates proliferation, migration, and differentiation, and is intrinsically involved in cardiovascular development [21–23]. Previous studies have confirmed that Wnt3a and Wnt5a contribute to human cardiac fibroblasts development [24]. Similarly, TCF participates in cardio pharyngeal development in Ciona. [25]. Interestingly, numerous studies have indicated that ncRNAs exert a marked effect on cardiac diseases via the Wnt signaling pathway. MiR-135b could stabilize β-catenin and thus activate Wnt signaling by suppressing Apc expression [26]. Knockdown of H19 efficiently suppressed proliferation and facilitated apoptosis in ox-LDL-treated human aorta VSMCs by blocking the Wnt/β-catenin pathway, thus alleviating intimal thickening [27]. Therefore, more research is needed to explore further associations between lncRNAs, miRNAs, and Wnt signaling in cardiac hypertrophy.

In the present study, we identified a new lncRNA, lncRNAA02Rik, which was upregulated in cardiac hypertrophy models in vivo and in vitro and appeared to function as an endogenous RNA to sponge miR-135a. Overexpression of lncRNAA02Rik and knockdown of miR-135a in myocardial cells produced the hypertrophic phenotype. Furthermore, TCF7 was demonstrated to be the direct target gene for miR-135a. Taken together, our study demonstrated that lncRNAA02Rik contributed to cardiac hypertrophy via sponging miR-135a and activating TCF7, a component of the Wnt signaling pathway.

## Results

### Inhibition of lncRNAA02Rik suppressed pressure overload-induced cardiac hypertrophy

**Fig. 1:**
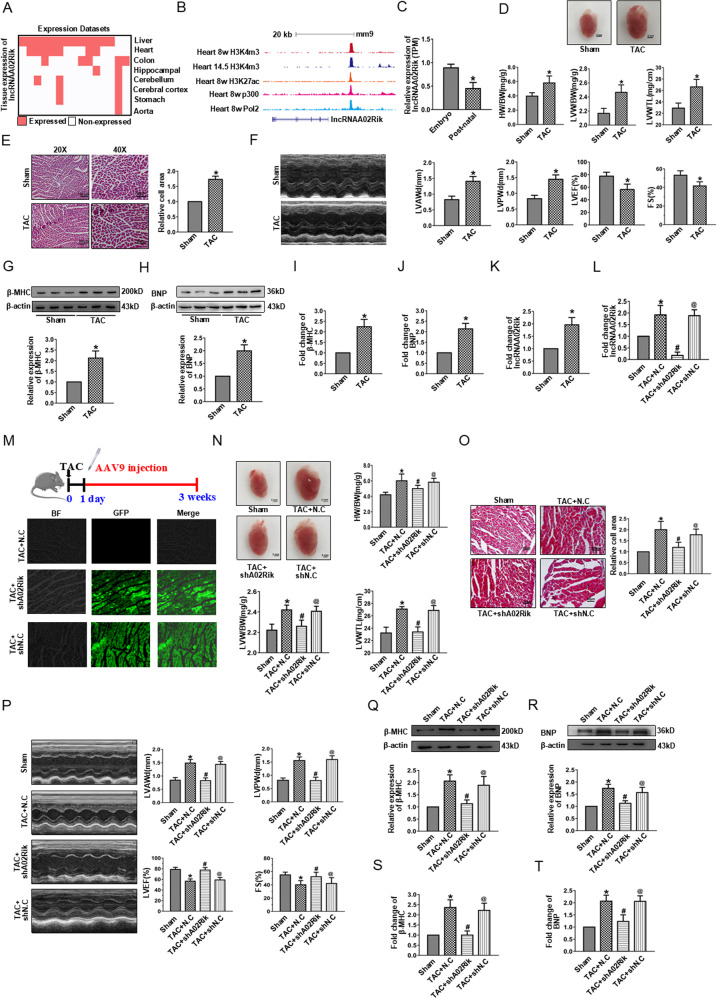
Knockdown of lncRNAA02Rik by shRNA attenuated cardiac hypertrophy in vivo. **A** The heatmaps of lncRNAA02Rik expression in eight tissues. **B** LncRNAA02Rik gene epigenomic signals in heart tissues. **C** The change in expression of lncRNAA02Rik following heart development. **D** HW/BW heart weight to body weight ratio, LVW/BW left ventricle weight to body weight ratio, LVW/TL left ventricle weight to tibial length ratio. **E** Representative images of HE staining of mouse heart sections. Bar = 100 μm for 20X. Bar = 50 μm for 40X. **F** Echocardiographic parameters in mouse hearts. **G**, **H** Western blot results of β-MHC and BNP protein expression. **I**, **J** mRNA levels of β-MHC and BNP. **K** LncRNAA02Rik expression in TAC models in vivo. **L** LncRNAA02Rik expression by shA02Rik in vivo. **M** Protocol for AAV9-shA02Rik/AAV9-shN.C injection. **N** Cardiac morphology. Bar: 2 mm. **O** Representative images of HE staining of mouse heart sections and statistical analysis of cell area. Bar: 50 μm. **P** Representative images of echocardiography and statistical results of echocardiographic parameters in mouse hearts. **Q**, **R** Western blot results of β-MHC and BNP protein expression. **S**, **T** mRNA levels of β-MHC and BNP. *P < 0.05 vs. Sham/Embryo group, ^*#*^*P* < 0.05 vs. TAC + N.C group, ^@^*P* < 0.05 vs. TAC + shA02Rik group, *n* = 6.

Our previous studies determined that three lncRNAs (4930473A02Rik, Gm15834, and Gm11508) were the potential risk factors in cardiac hypertrophy [28]. Here, based on the tissue expression patterns of lncRNAs in the MGI database, we found that lncRNAA02Rik was also a heart-specific expressed transcript (Fig. 1A). We then investigated the transcriptional activity of lncRNAA02Rik in the heart based on UCSC ChIP-seq tracks. As a result, lncRNAA02Rik promoter regions were occupied by abundant ChIP-seq signals, such as H3K4me3, H3K27ac, P300, and Pol2 (Fig. 1B), suggesting that lncRNAA02Rik had strong transcriptional activity. Moreover, we investigated the expression tendency of lncRNAA02Rik in heart development using ENCODE expression data. Results showed that lncRNAA02Rik was activated during the embryo period and was silenced along with heart development (Fig. 1C). Previous studies have validated that hypertrophy genes show higher expression levels during the early heart development period rather than during the adult period. Prior to expression, 3-weeks post-TAC surgery was first performed. The results showed a significantly larger proportion of heart tissue (Fig. 1D), increased cell area (Fig. 1E), and impaired cardiac function in the TAC group (Fig. 1F). Meanwhile, the increased protein and RNA levels of cardiac hypertrophy marker proteins (β-MHC and BNP) were found in TAC groups (Fig. 1G–J). Thereafter, lncRNAA02Rik expression was detected, which had been proven to be significantly enriched in hypertrophic heart tissue (Fig. 1K), suggesting that lncRNAA02Rik might be associated with the regulation of cardiac hypertrophy development.

To clarify whether the upregulation of lncRNAA02Rik contributed to cardiac hypertrophy in the mice model, we knocked down lncRNAA02Rik in mice by injecting adeno-associated virus-9 carrying a lncRNAA02Rik-specific shRNA (AAV9-shA02Rik) through the tail vein. After TAC surgery at 3 weeks, significant AAV9-shA02Rik expression was observed in cardiac tissues (Fig. 1L, M). Notably, relative to the TAC group, treatment with AAV9-shA02Rik decreased the ratios of HW/BW, LVW/BW, and LVW/TL (Fig. 1N) and reduced cardiomyocyte surface area (Fig. 1O). Furthermore, there was a decreasing trend in LVAWd and LVPWd, whereas there was an increase in LVEF and FS due to the effect of AAV9-shA02Rik, relative to the TAC group (Fig. 1P). Furthermore, AAV9-shA02Rik transfection inhibited protein upregulation and mRNA levels of cardiac hypertrophy markers BNP and β-MHC (Fig. 1Q–T). These in vivo results indicated that lncRNAA02Rik knockdown could inhibit cardiac hypertrophy.

### Inhibition of lncRNAA02Rik prevented cardiomyocyte hypertrophy in vitro

**Fig. 2:**
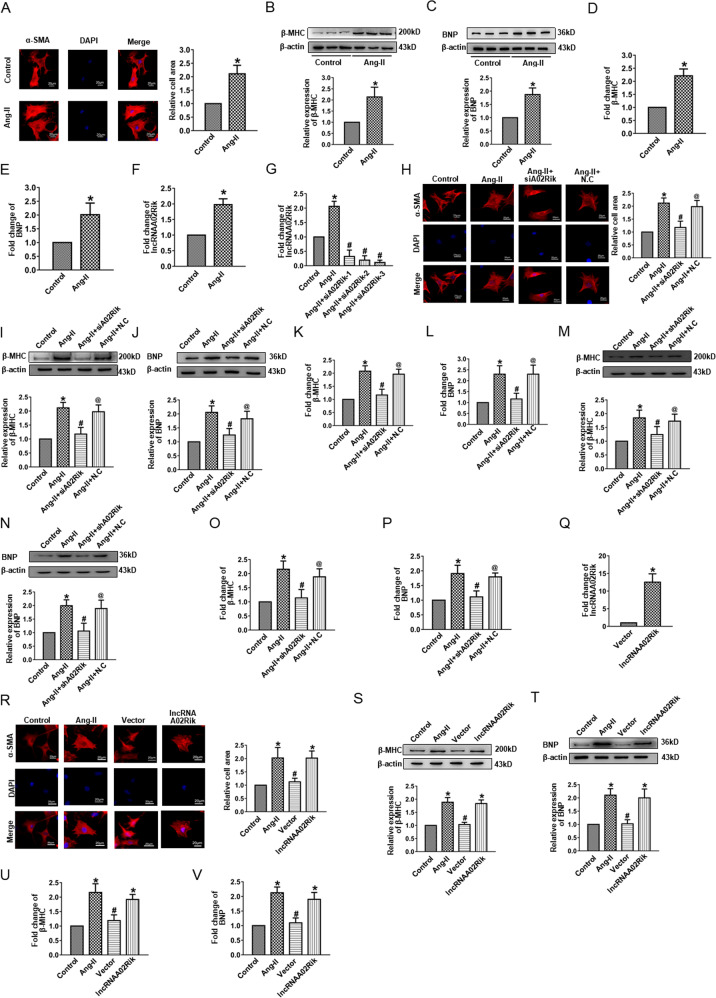
LncRNAA02Rik contributed to cardiac hypertrophy in vitro. **A** Immunostaining of α-SMA in cardiomyocytes. Bar: 20 μm. **B**, **C** Western blot results of β-MHC and BNP protein expression. **D**, **E** mRNA levels of β-MHC and BNP. **F** LncRNAA02Rik expression in Ang-II-induced models in vitro. **G** Knockdown of lncRNAA02Rik by siRNAs. **H** Immunofluorescence staining of α-SMA in cardiomyocytes demonstrated that lncRNAA02Rik knockdown decreased the cell surface area. Bar: 20 μm. **I**, **J** Western blot results of β-MHC and BNP protein expression in cardiomyocytes by siA02Rik. **K**, **L** mRNA levels of β-MHC and BNP in cardiomyocytes by siA02Rik. **M**, **N** β-MHC and BNP protein expression by shA02Rik in vitro. **O**, **P** The mRNA levels of β-MHC and BNP by shA02Rik in vitro. **Q** Forced expression of lncRNAA02Rik with a lncRNAA02Rik overexpression plasmid. **R** Immunofluorescence staining of α-SMA in cardiomyocytes demonstrated that enhanced lncRNAA02Rik expression promoted the cell surface area. Bar: 20 μm. **S**, **T** Western blot results of β-MHC and BNP protein expression in cardiomyocytes by lncRNAA02Rik overexpression. **U**, **V** mRNA levels of β-MHC and BNP in cardiomyocytes by lncRNAA02Rik overexpression. *P < 0.05 vs. Control/Vector group, ^#^*P* < 0.05 vs. Ang-II/lncRNAA02Rik group, ^@^*P* < 0.05 vs. Ang-II + siA02Rik/Ang-II + shA02Rik group, *n* = 6.

To determine the role of lncRNAA02Rik in the in vitro model, Ang-II was used to induce cardiac hypertrophy. In order to check if we really excluded fibroblasts and myofibroblasts from cardiomyocytes, a specific marker for cardiomyocytes (cardiac troponin T) was used to stain cardiomyocytes. Results showed that the cells were cardiomyocytes stained by cardiac troponin T (Supplementary Fig. 1A). The cell area was demonstrably enlarged (Fig. 2A), and the protein and mRNA expression levels of BNP and β-MHC were significantly upregulated upon Ang-II treatment (Fig. 2B–E). Meanwhile, lncRNAA02Rik expression was detected, which was increased in the Ang-II group (Fig. 2F).

To confirm the action of lncRNAA02Rik in cardiomyocyte hypertrophy, a loss-of-function experiment was undertaken. As displayed in Fig. 2G, siA02Rik-3 showed the most potent inhibitory effect on lncRNAA02Rik among the three siRNAs. Notably, silencing lncRNAA02Rik by siRNA targeting lncRNAA02Rik gene downregulated cardiomyocyte surface area (Fig. 2H). Meanwhile, lncRNAA02Rik knockdown significantly alleviated the protein and mRNA level of β-MHC and BNP induced by Ang-II (Fig. 2I–L). Additionally, similar results were also obtained by shA02Rik (Fig. 2M–P). The above data suggested that lncRNAA02Rik deficiency protected neonatal cardiomyocytes from hypertrophic responses. Furthermore, we employed the pcDNA3.1 plasmid to overexpress lncRNAA02Rik in cardiomyocytes. As illustrated in Fig. 2Q, lncRNAA02Rik showed a significant increase compared with the vector plasmid group. This result demonstrated that we successfully overexpressed lncRNAA02Rik in cardiomyocytes. Additionally, we observed that lncRNAA02Rik overexpression increased cell surface area (Fig. 2R) and promoted β-MHC and BNP mRNA and protein expression (Fig. 2S–V). The above results indicated that lncRNAA02Rik contributed to cardiac hypertrophy in vitro.

### miR-135a was the potential target of lncRNAA02Rik

**Fig. 3:**
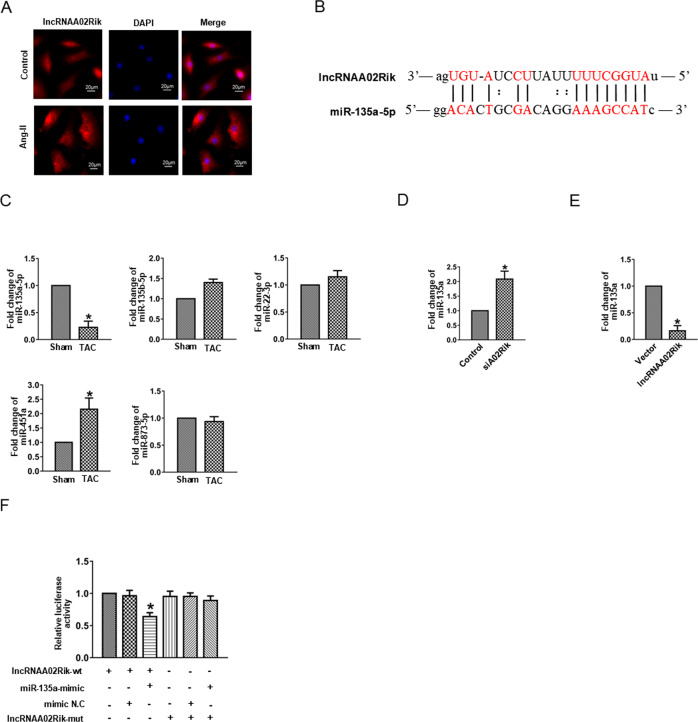
miR-135a was the target of lncRNAA02Rik. **A** Subcellular localization detection of lncRNAA02Rik. Bar: 20 μm. **B** The binding sites between lncRNAA02Rik and miR-135a were determined using miRanda software. **C** Relative expression of miR-135a-5p, miR-135b-5p, miR-22-3p, miR-451a, and miR-873-5p. **D** Expression level of miR-135a in cardiomyocytes transfected with siA02Rik. **E** Expression level of miR-135a in cardiomyocytes transfected with the lncRNAA02Rik plasmid. *P < 0.05 vs. Control/Vector/Sham group, *n* = 6. **F** Luciferase reporter gene assay of lncRNAA02Rik and miR-135a. *P < 0.05 vs. lncRNAA02Rik-wt group, *n* = 3.

Evidence has shown that lncRNAs could affect miRNA function by acting as a ceRNA. Accordingly, we determined lncRNAA02Rik location using RNA fluorescence in situ hybridization (FISH) assays and found that most lncRNAA02Rik was located in the cytoplasm (Fig. 3A). Moreover, miR-135a was identified as one of the potential targets of lncRNAA02Rik using miRanda software and contained putative binding sites of lncRNAA02Rik (Fig. 3B). Meanwhile, miR-135a expression was differentially decreased in pressure overload-induced hypertrophic hearts (Fig. 3C). Additionally, studies have documented that miR-135a expression was altered by lncRNAA02Rik overexpression or knockdown (Fig. 3D, E). To acquire more direct evidence of the interaction between lncRNAA02Rik and miR-135a, as shown in Fig. 3F, it was observed that miR-135a decreased the luciferase activity of wild-type lncRNAA02Rik. In contrast, miR-135a did not affect mutant-type lncRNAA02Rik, indicating a direct binding relationship between lncRNAA02Rik and miR-135a.

### miR-135a protected against cardiac hypertrophy by targeting TCF7

**Fig. 4:**
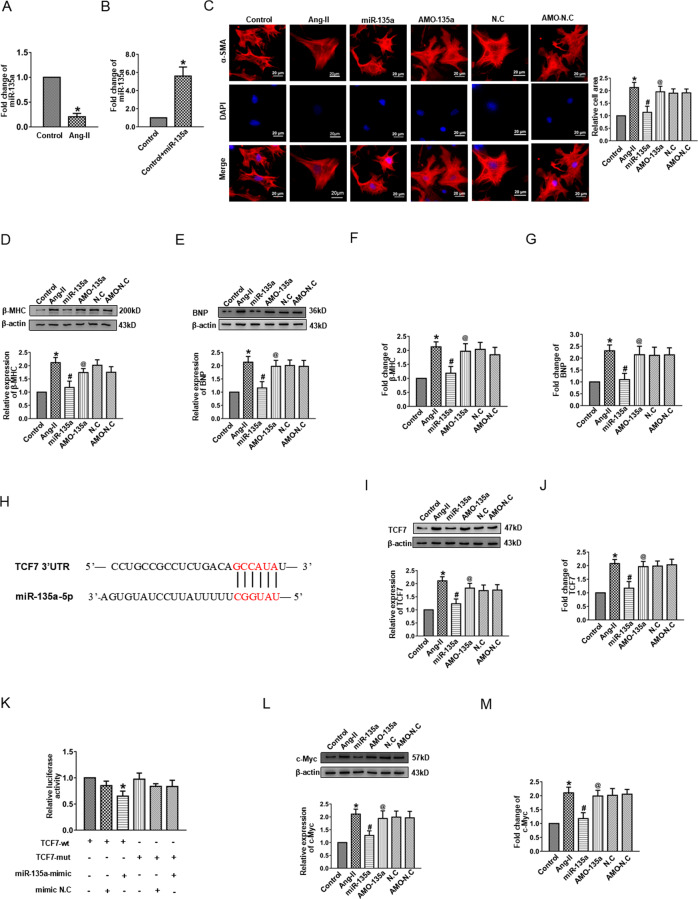
miR-135a inhibited cardiac hypertrophy by targeting TCF7. **A** The expressions of miR-135a in cardiac hypertrophy models in vitro. **B** Expression level of miR-135a in cardiomyocytes transfected with scramble or miR-135a mimics. **C** Immunostaining of α-SMA in cardiomyocytes transfected with miR-135a mimics. Bar: 20 μm. **D**, **E** Western blot results of β-MHC and BNP protein expression in cardiomyocytes transfected with miR-135a mimics. **F**, **G** mRNA levels of β-MHC and BNP in cardiomyocytes transfected with miR-135a mimics. **H** The binding sites of miR-135a and TCF7 were determined using TargetScan software. **I** Western blot results of TCF7 protein expression by miR-135a mimics. **J** The mRNA level of TCF7 in cardiomyocytes by miR-135a mimics. **K** Luciferase reporter activities of chimeric vectors carrying the luciferase gene and a fragment of TCF7 3′-UTR containing the binding sites of miR-135a. *P < 0.05 vs. TCF7-wt group, *n* = 3. **L** Western blot results of c-Myc protein expression by miR-135a mimics. **M** The mRNA level of c-Myc in cardiomyocytes by miR-135a mimics. *P < 0.05 vs. Control group, ^#^*P* < 0.05 vs. Ang-II group, ^@^*P* < 0.05 vs. miR-135a group, *n* = 6.

Following the above results, miR-135a expression was detected and was significantly downregulated in Ang-II-treated neonatal cardiomyocytes (Fig. 4A). These findings raised the possibility that miR-135a might be associated with the development of cardiac hypertrophy. Subsequently, we successfully overexpressed miR-135a in cardiomyocytes (Fig. 4B) to examine its effect on cardiac hypertrophy. Overexpression of miR-135a dramatically diminished the enlarged cell surface areas as well as protein and mRNA levels of BNP and β-MHC induced by Ang-II. However, these effects could be reversed by AMO-135a (Fig. 4C–G). These results suggested that miR-135a was involved in regulating of cardiac hypertrophy.

Using a bioinformatics approach, we then found that TCF7 mRNA 3′-UTRs comprised ‘seed’ sequences and flanking nucleotides matching miR-135a (Fig. 4H). Therefore, the impact of miR-135a on TCF7 expression using the miR-135a mimic and inhibitor was analyzed. Western blot results demonstrated that TCF7 protein expression was inhibited by overexpressing miR-135a and was promoted by AMO-135a (Fig. 4I). Similarly, the mRNA level of TCF7 change was consistent with western blot results (Fig. 4J). To investigate whether TCF7 was directly inhibited by miR-135a, we prepared luciferase constructs carrying the TCF7 3′-UTR. Results showed that the luciferase activity of wild-type TCF7 was decreased sharply by the miR-135a mimic. However, the activity of the mutant-type TCF7 was almost unchanged upon miR-135a overexpression (Fig. 4K). These data revealed a significant negative correlation between TCF7 and miR-135a and confirmed that TCF7 was the potential target gene for miR-135a. Since TCF7 is an important component of the Wnt signaling pathway, we next sought to determine whether miR-135a could affect the activity of Wnt signaling. Ultimately, we found that the protein and mRNA levels of the downstream target gene of the Wnt signaling pathway, c-Myc, were reduced by miR-135a overexpression, and the effect was restored by AMO-135a (Fig. 4L–M). Overall, these data indicated that miR-135a suppressed cardiac hypertrophy by directly binding to TCF7.

### TCF7, a member of Wnt signaling, promoted cardiac hypertrophy

**Fig. 5:**
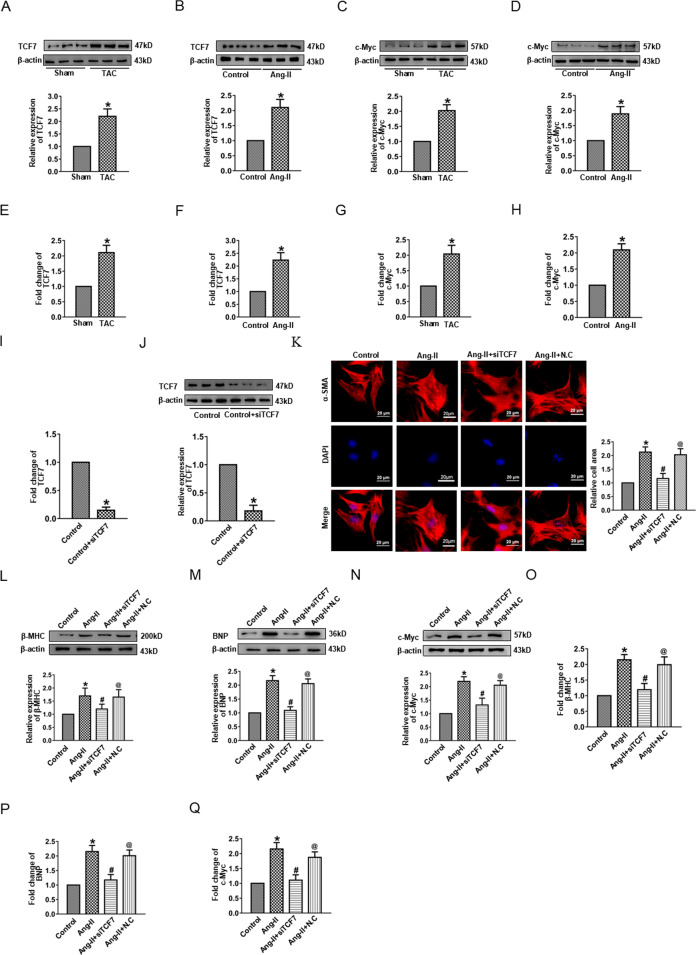
The effect of TCF7 on cardiac hypertrophy. **A**, **B** Western blot results of TCF7 protein expression. **C**, **D** Western blot results of c-Myc protein expression. **E**, **F** mRNA level of TCF7. **G**, **H** mRNA level of c-Myc. **I** mRNA level of TCF7 in cardiomyocytes by siTCF7. **J** TCF7 protein expression in cardiomyocytes by siTCF7. **K** Immunostaining of α-SMA in cardiomyocytes transfected with siTCF7. Bar: 20 μm. **L**, **M** Western blot results of β-MHC and BNP protein expression in cardiomyocytes by siTCF7. **N** Western blot results of c-Myc protein expression in cardiomyocytes by siTCF7. **O**, **P** mRNA levels of β-MHC and BNP in cardiomyocytes by siTCF7. **Q** mRNA level of c-Myc in cardiomyocytes by siTCF7. *P < 0.05 vs. Sham/Control group, ^#^*P* < 0.05 vs. Ang-II group, ^@^*P* < 0.05 vs. Ang-II + siTCF7 group, *n* = 6.

Accumulating evidence indicates that the Wnt signaling pathway propagates the initiation and progression of cardiac diseases. However, TCF7, an important member of the Wnt signaling pathway, has an ambiguous relationship with cardiac hypertrophy. Thus, we sought to confirm whether TCF7 was involved in hypertrophic models. Elevated TCF7 protein expressions were confirmed in mouse heart tissue after TAC and in cardiomyocytes treated with Ang-II compared with sham or control group (Fig. 5A, B). Meanwhile, the protein level of c-Myc, the downstream target of TCF7, was increased consistently (Fig. 5C, D). The same changes in TCF7 and c-Myc RNA levels were also observed in in vivo and in vitro groups (Fig. 5E–H). Thereafter, we used siRNA to silence TCF7 (Fig. 5I, J). Notably, we found that silencing TCF7 decreased the cell surface area (Fig. 5K), expressions of hypertrophy-related markers β-MHC (Fig. 5L), and BNP (Fig. 5M), as well as the c-Myc protein level (Fig. 5N) following Ang-II treatment. Similarly, the mRNA levels were also reduced by siTCF7 (Fig. 5O–Q). These data revealed that TCF7 acted as a component of Wnt signaling to promote the occurrence of cardiac hypertrophy.

### LncRNAA02Rik had a positive effect on Wnt signaling

**Fig. 6:**
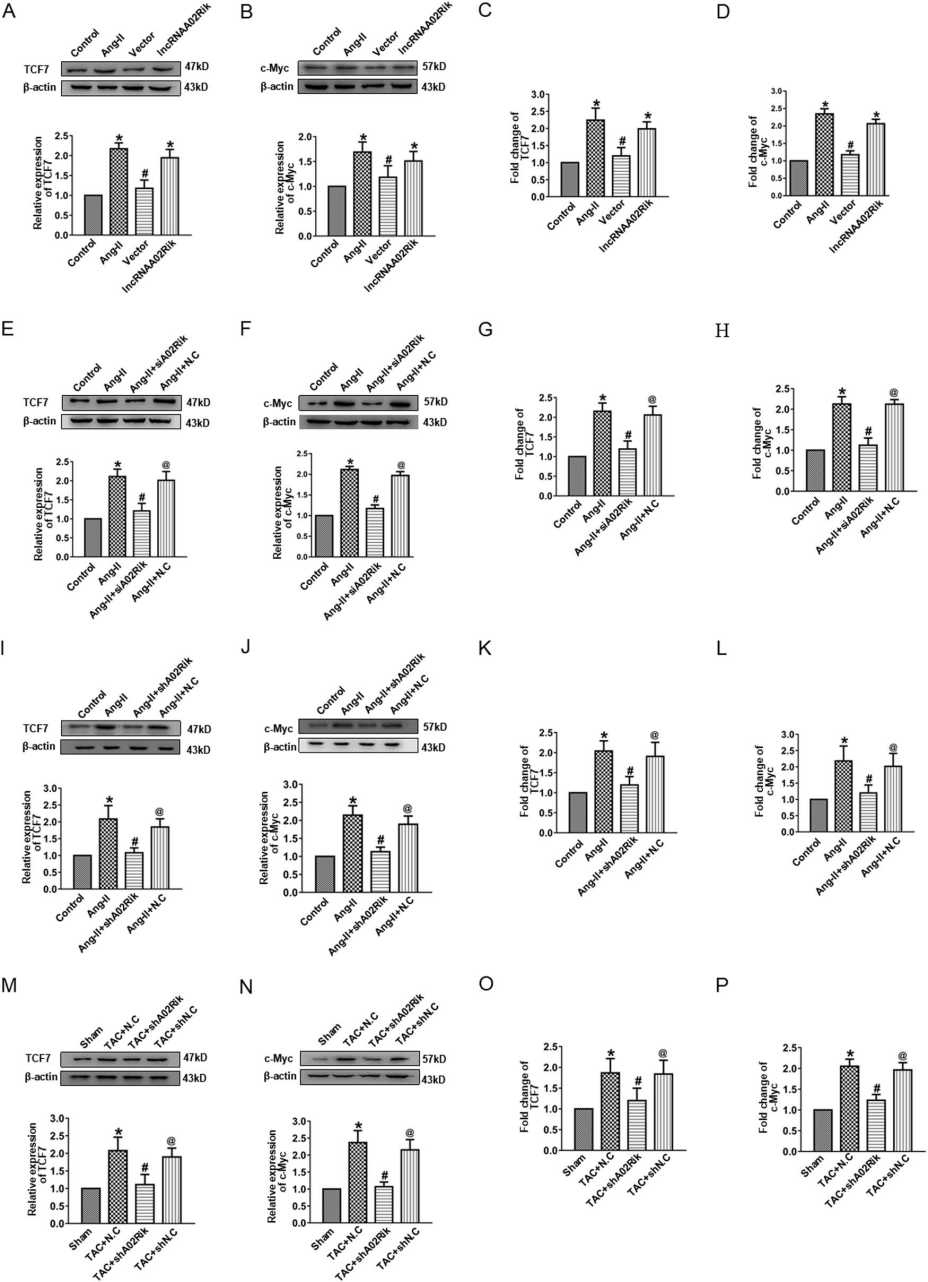
The effect of lncRNAA02Rik on Wnt signaling. **A**, **B** Western blot results of TCF7 and c-Myc protein expression by lncRNA02Rik overexpression. **C**, **D** mRNA levels of TCF7 and c-Myc by lncRNA02Rik overexpression. **E**, **F** Western blot results of TCF7 and c-Myc protein expression by siA02Rik. **G**, **H** mRNA levels of TCF7 and c-Myc by siA02Rik. **I**, **J** Western blot results of TCF7 and c-Myc protein expression by shA02Rik in vitro. **K**, **L** mRNA levels of TCF7 and c-Myc by shA02Rik in vitro. **M**, **N** Western blot results of TCF7 and c-Myc protein expression by shA02Rik in vivo. **O**, **P** mRNA levels of TCF7 and c-Myc by shA02Rik in vivo. *P < 0.05 vs. Sham/Control group, ^#^*P* < 0.05 vs. TAC + N.C/Ang-II/lncRNAA02Rik group, ^@^*P* < 0.05 vs. Ang-II + siA02Rik/Ang-II + shA02Rik/TAC + shA02Rik group, *n* = 6.

To assess the effect of lncRNAA02Rik on Wnt signaling, we overexpressed lncRNAA02Rik in cardiomyocytes and found that the protein and mRNA levels of TCF7 and c-Myc were increased (Fig. 6A–D). Consistently, lncRNAA02Rik knockdown by siRNA/shRNA led to decreased protein and mRNA levels of both TCF7 and c-Myc (Fig. 6E–L). Meanwhile, we found that lncRNAA02Rik knockdown in TAC mice also resulted in the downregulation of the activity of TCF7 and c-Myc (Fig. 6M–P). These results indicated that lncRNAA02Rik positively mediated Wnt signaling activity.

### LncRNAA02Rik contributed to cardiac hypertrophy via miR-135a/TCF7 axis

**Fig. 7:**
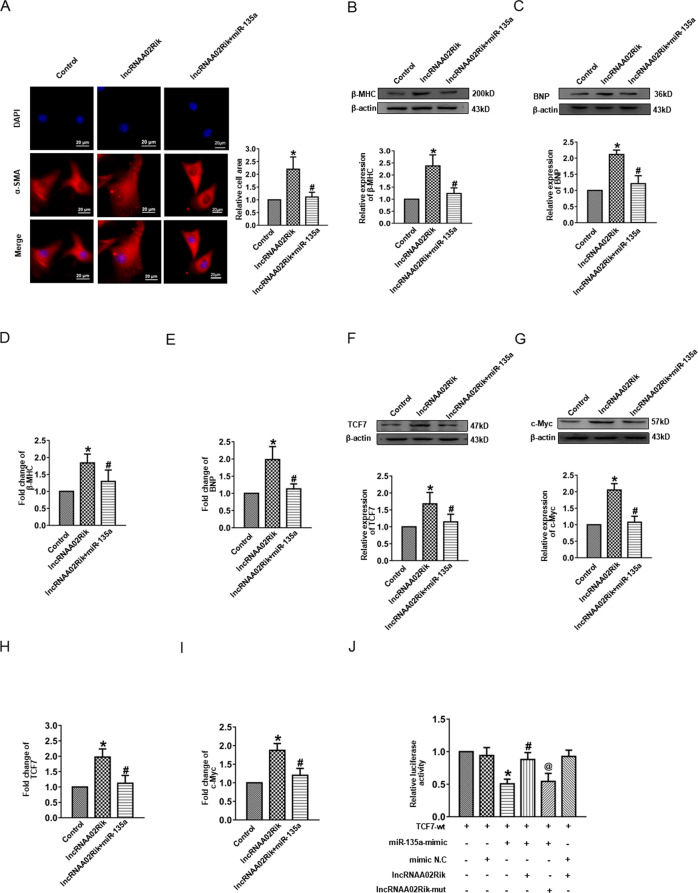
The relationship between lncRNAA02Rik, miR-135a, and TCF7. **A** Immunostaining of α-SMA by co-transfection of lncRNAA02Rik and miR-135a in cardiomyocytes. **B**, **C** Western blot results of β-MHC and BNP protein expression in cardiomyocytes by co-transfection. **D**, **E** mRNA levels of β-MHC and BNP in cardiomyocytes by co-transfection. **F**, **G** Western blot results of TCF7 and c-Myc protein expression in cardiomyocytes by co-transfection. **H**, **I** mRNA levels of TCF7 and c-Myc in cardiomyocytes by co-transfection. *P < 0.05 vs. Control group, ^#^*P* < 0.05 vs. lncRNA02Rik group, *n* = 6. **J** The luciferase reporter gene was used to validate the regulatory interactions among lncRNAA02Rik, miR-135a, and TCF7. *P < 0.05 vs. TCF7-wt group, ^#^*P* < 0.05 vs. TCF7-wt+miR-135a-mimic group, ^@^*P* < 0.05 vs. TCF7-wt + miR-135a-mimic + lncRNAA02Rik group, *n* = 3.

The above results demonstrated that lncRNAA02Rik promoted cardiac hypertrophy. Moreover, miR-135a was a direct target for lncRNAA02Rik, and it inhibited cardiac hypertrophy by suppressing TCF7-mediated Wnt pathway activity. Therefore, we hypothesized that lncRNAA02Rik sponged miR-135a and weakened TCF7 suppression, leading to excessive Wnt pathway activity, ultimately causing cardiac hypertrophy. We then co-transfected lncRNAA02Rik and miR-135a into cardiomyocytes and observed that miR-135a significantly altered cardiomyocyte surface area induced by lncRNAA02Rik (Fig. 7A). Furthermore, the protein and mRNA levels of hypertrophic biomarkers (β-MHC and BNP) were lower in the co-transfected group than in the lncRNAA02Rik only group (Fig. 7B–E), indicating that lncRNAA02Rik promoted cardiac hypertrophy by sponged miR-135a. After co-transfecting lncRNAA02Rik and miR-135a into cardiomyocytes, miR-135a could reverse the effect of lncRNAA02Rik on TCF7 and c-Myc (Fig. 7F–I). Notably, the luciferase result demonstrated that miR-135a could diminish the luciferase activity of TCF7, while lncRNAA02Rik could reverse this effect. However, lncRNAA02Rik did not affect TCF7 activity (Fig. 7J). Collectively, these results confirmed that lncRNAA02Rik promoted cardiac hypertrophy via the miR-135a/TCF7 pathway.

## Discussion and conclusion

Unlike an athlete’s heart, pathological cardiac hypertrophy is triggered by pressure overload or diseases, leading to an increase in cell size rather than enhanced pumping ability. Although this problem has been extensively studied, major challenges remain.

In the present study, we reported for the first time that lncRNAA02Rik acted as a regulator of cardiac hypertrophy. We started our research by detecting enhanced lncRNAA02Rik expression in hypertrophic mouse hearts and cardiomyocytes. Meanwhile, this upregulation aggravated the hypertrophic phenotype both in vivo and in vitro. Furthermore, miR-135a functioned as an anti-hypertrophy miRNA by sponging lncRNAA02Rik. In addition, TCF7 served as the target for miR-135a. Moreover, siTCF7 had a beneficial role in cardiac hypertrophic responses. Collectively, our study demonstrated that lncRNAA02Rik exerted its hypertrophic effect through the miR-135a/TCF7 axis.

More recently, a growing number of lncRNAs have been confirmed to be responsible for cardiac diseases [29, 30]. LncRNAA02Rik, a novel member of lncRNAs, was increased in the cardiac hypertrophy model-a finding that triggered the present study. Through gain- and loss-of-function experiments, artificial lncRNAA02Rik overexpression in normal cardiomyocytes significantly enlarged cell area and enhanced the protein and mRNA levels of hypertrophic markers (BNP and β-MHC). In contrast, silencing lncRNAA02Rik in hypertrophic neonatal mouse ventricular myocytes and TAC-induced hypertrophic mouse hearts markedly ameliorated hypertrophic heart function, decreased cell surface area upregulation and markedly reduced the mRNA and protein levels of BNP and β-MHC. These results indicated that lncRNAA02Rik might have a pro-hypertrophy role, and further detailed studies were warranted to address this phenomenon.

Accumulating evidence has shown that lncRNAs could function as ceRNA to indirectly exert biological functions in various diseases, including cardiac hypertrophy [31, 32]. For example, the lncRNAH19 functioned as a ceRNA to mediate cardiac fibrosis [33]. To explore whether lncRNAA02Rik mediated cardiac hypertrophy by acting as ceRNA, we first conducted bioinformatics analysis using the miRanda software and determined that miR-135a contained potential binding sequences for lncRNAA02Rik. After the prediction, RNA FISH was performed to detect the subcellular localization of lncRNAA02Rik, and the results showed that most lncRNAA02Rik was located in the cytoplasm. In particular, forcing the overexpression or silence of lncRNAA02Rik could weaken or promote miR-135a expression, respectively. Additionally, co-transfection of lncRNAA02Rik and miR-135a could reverse the pro-hypertrophic effect of lncRNAA02Rik. At the same time, luciferase results indicated that lncRNAA02Rik could directly bind to miR-135a. Therefore, we concluded that lncRNAA02Rik promoted cardiac hypertrophy by competitively sponging miR-135a. Next, we investigated the effect of miR-135a on cardiac hypertrophy.

In general, miRNA-mediated gene expression and the regulation of further downstream signaling events exerted an appreciable influence on cardiac hypertrophy progression [34]. However, the role of miR-135a in cardiac hypertrophy has not been reported. In the present study, miR-135a was revealed to be significantly inhibited, both in the TAC-induced mouse heart and cultured mouse myocardial cells following 24 h of Ang-II treatment. We then found that forcing miR-135a overexpression could significantly repress the myocardial cell area and the expression of BNP and β-MHC proteins in vitro. On the contrary, the anti-miR-135a significantly enhanced their expressions in cardiac hypertrophy. Furthermore, to identify the direct target for miR-135a, TargetScan software was used to predict the downstream genes of miR-135a. We found that TCF7 was the most likely target gene for the miR-135a. The luciferase activity and TCF7 protein expression results confirmed that TCF7, a central component of the Wnt signaling pathway whose downstream target was c-Myc, was a direct target for miR-135a. In this study, TCF7 and c-Myc levels were significantly increased in TAC-induced mouse hearts and Ang-II-treated cultured mouse myocardial cells. Furthermore, silencing TCF7 could significantly diminish the cardiomyocyte area and decrease BNP and β-MHC protein levels and mRNA expression. Taken together, the data suggested that the miR-135a protected against cardiac hypertrophy by inhibiting TCF7 expression and then blocking the Wnt signaling pathway. Moreover, the luciferase experiment demonstrated that lncRNAA02Rik sponging miR-135a could abolish the derogation of the target gene TCF7 by miR-135a. Furthermore, co-transfection of lncRNAA02Rik and miR-135a could ameliorate the pro-hypertrophic effect of lncRNAA02Rik. Therefore, lncRNAA02Rik promoted cardiac hypertrophy through the miR-135a/TCF7 signaling pathway.

Taken together, our study revealed that lncRNAA02Rik, as a critical pro-hypertrophic lncRNA, could promote cardiac hypertrophy via the miR-135a/TCF7 signaling pathway, implying that lncRNAA02Rik could be considered as a novel therapeutic target. Accordingly, siA02Rik or other forms of the lncRNAA02Rik inhibitor could be developed into novel therapeutic agents for treating cardiac hypertrophy.

## Materials and methods

### Bioinformatics analysis of lncRNAA02Rik

To observe the expression patterns of lncRNAA02Rik in mouse hearts and other tissues, we first downloaded the expression profile of lncRNAA02Rik from the MGI database (http://www.informatics.jax.org/). Then, we classified the expression data into “expressed” or “non-expressed” groups based on the expression thresholds of the MGI database and exhibited these in a heatmap. We also observed the transcriptional activity of lncRNAA02Rik in the heart. Briefly, we downloaded ChIP-seq data (histone: H3K4me3 and H3K27ac; Co-factor: P300 and Pol2) of the heart from the UCSC table browsers (http://genome.ucsc.edu/), and data visualization was conducted using the IGV browser with mm9 as the reference genome. In addition, we also downloaded heart development expression data from the ENCODE database (https://www.encodeproject.org/).

### Cardiac hypertrophy induced by pressure overload in vivo

All the animal experimental procedures were performed following the Guide for the Care and Use of Laboratory Animals published by the US NIH (publication, 8th Edition, 2011), and approved by the Experimental Animal Ethics Committee of Harbin Medical University-Daqing. Briefly, C57BL/6 mice of 22–24 g (8–10 weeks old) were anesthetized by intraperitoneal injection with sodium pentobarbital (30 mg/kg, i.p.). To visualize the aortic arch, under aseptic conditions, a 2–3 mm longitudinal incision was performed in the proximal sternum. An overlaying blunted 27-gauge needle and a 6-0 silk suture were then used for transverse aorta constriction; the needle was quickly removed to make the constriction. Mice received meloxicam (1.6 mg/kg, s.c.) immediately after surgery and again 12 h postoperatively. Mice were kept warm under a heat lamp and monitored until they were awake and ambulatory, at which time they were returned to their home cages. After 3 weeks, echocardiography analysis was conducted to evaluate cardiac function, including left ventricular posterior wall end-diastolic diameter (LVPWd), left ventricular anterior wall end-diastolic diameter (LVAWd), left ventricular ejection fraction (LVEF), and fractional shortening (FS). Finally, the mice were sacrificed to examine the heart weight (HW), body weight (BW), left ventricular weight (LVW), and tibial length (TL) [8].

### Neonatal mouse cardiomyocyte culture

Neonatal mouse cardiomyocytes were harvested as previous study [35–37]. Briefly, hearts were obtained from C57BL/6 mice (1 to 3 days old) and repeatedly rinsed with ice-cold phosphate-buffered saline solution. Thereafter, ventricular tissues were minced with scissors and enzymatically dissociated using 0.25% trypsin at 37 °C, and pooled cell suspensions were centrifuged and then resuspended in Dulbecco’s modified Eagle’s medium with 10% fetal bovine serum. The suspension was coated on the culture bottle for 90 min, allowing the fibroblasts to attach preferentially to the bottom of the bottle. Weak-adherent and non-adherent cells were regarded as cardiomyocytes and transferred to a new culture bottle for further experiments, and 0.01 nmol/L 5-Bromo-2-deoxydriuine was added to exclude the cardiac fibroblasts. Finally, to induce hypertrophy, angiotensin-II (Ang-II) was added to the cardiomyocyte at a concentration of 100 nmol/L for 24 h.

### Western blotting

Myocardial cells and heart tissue were lysed in cold radioimmunoprecipitation assay buffer. A bicinchoninic acid protein assay was used to quantify protein samples. Identical quantities of protein samples were separated by SDS-PAGE and transferred onto a nitrocellulose membrane. Thereafter, the membrane was blocked with 5% skim milk at room temperature for 2 h, followed by incubation with the specific primary antibody at 4 °C overnight: anti-BNP antibody (1:1,000 dilution, sc-271185, Santa Cruz), anti-β-MHC antibody (1:2,000 dilution, SAB2106550, Sigma), anti-TCF7 antibody (1:300 dilution, A01315-2, Boster), anti-c-Myc antibody (1:300 dilution, BM4042, Boster), and anti-β-actin antibody (1:2000 dilution, TA-09, ZSGB-BIO). β-actin was used as a loading control. After washing thrice for 10 min each time in Tris-buffered saline with Tween (TBST), the membrane was incubated with the secondary antibody at room temperature for 1 h and washed again in TBST. Subsequently, the bands were scanned using an Odyssey Imaging System (LI-COR Biosciences, Lincoln, NE, USA).

### Hematoxylin-eosin (HE) staining

After anesthesia, the hearts were quickly removed and immersed in 4% paraformaldehyde solution for 24 h. Next, the tissue was cut into 5-μm-thick cross-sectional slices. These slices were stained with HE to evaluate histopathology. Photographs were captured using an Olympus BX60 microscope (Olympus Optical, Tokyo, Japan), and cell areas were calculated using image analysis software (Image-Pro Plus 6.0 software).

### Immunofluorescence staining

The cells on coverslips were fixed with 4% paraformaldehyde for 15 min and permeabilized with 0.2% Triton X-100 for 15 min. Thereafter, goat serum was used to block cells for 30 min at room temperature. α-Smooth muscle actin (α-SMA) antibody (1:200 dilution, #19245, Cell Signaling) and cardiac troponin T antibody (1:100 dilution, A4914, ABclonal) were added, followed by incubation at 4 °C overnight [38, 39]. The second antibody was then added, followed by incubation in the dark for 1 h. The slides were observed under a fluorescence microscope (Leica, Heidelberg, Germany), and cell areas were calculated using image analysis software (Image-Pro Plus 6.0 software).

### Quantitative real-time polymerase chain reaction (qPCR)

TRIzol was used to extract the total RNA from the heart tissues of mice or cultured cells following the manufacturer’s protocol. qPCR was performed using a LightCycler^®^ 480 system (Roche, Basel, Switzerland) with SYBR Green I Master Mix (Roche). Each reaction system consisted of 20 ng cDNA. After a 40-cycle reaction, amplification was used to calculate the CT value (ΔCT) of target genes and the difference between the ΔCT of those genes and the 18 s, U6, or GAPDH gene. In addition, the 2^−ΔΔCT^ equation was used to determine the relative amount of lncRNA, miRNA, or mRNA in specific target genes.

The sequences of primers were as follows:

lncRNAA02Rik:

Forward: TGTCCATTCTGAGGGTTCTG

Reverse: ACGATTGAGGACTTCTGGC

miR-135a-5p reverse transcriptase primer:

CTCAACTGGTGTCGTGGAGTCGGCAATTCAGTTGAGTCACATAG

miR-135a-5p:

Forward: CTGGTAGGTATGGCTTTTTATTC

Reverse: TCAACTGGTGTCGTGGAGTC

miR-22-3p reverse transcriptase primer:

CTCAACTGGTGTCGTGGAGTCGGCAATTCAGTTGAGACAGTTCT

miR-22-3p:

Forward: ACACTCCAGCTGGGAAGCTGCCAGTTGAAG

Reverse: GGTGTCGTGGAGTCGGCAA

miR-135b-5p reverse transcriptase primer:

GTCGTATCCAGTGCGTGTCGTGGAGTCGGCAATTGCACTGGATACGACTCACAT

miR-135b-5p:

Forward: GGTATGGCTTTTCATTCCT

Reverse: CAGTGCGTGTCGTGGAGT

miR-451a reverse transcriptase primer:

GTCGTATCCAGTGCAGGGTCCGAGGTATTCGCACTGGATACGACACGCAA

miR-451a:

Forward: ACACTCCAGCTGGGAAACCGTTACCATTACT

Reverse: CTGGTGTCGTGGAGTCGGCAA

miR-873-5p reverse transcriptase primer:

GTCGTATCCAGTGCAGGGTCCGAGGTATTCGCACTGGATACGACGTCAAA

miR-873-5p:

Forward: GCAGGAACTTGTGAG

Reverse: GTGCAGGGTCCGAGGT

β-MHC:

Forward: TATCGATGACCTGGAGCTGA

Reverse: AGTATTGACCTTGTCTTCCTC

BNP:

Forward: ACAGAAGCTGCTGGAGCTGA

Reverse: CCGATCCGGTCTATCTTGTG

GAPDH:

Forward: ACAGCAACAGGGTGGTGGAC

Reverse: TTTGAGGGTGCAGCGAACTT

TCF7:

Forward: TCGGGTGTGGAGAAGACTGGCAT

Reverse: CTGGCTGATGTCCGCTGGTG

c-Myc:

Forward: CGTTGGAAACCCCGCAGACA

Reverse: GATATCCTCACTGGGCGCGG

U6:

Forward: CGCTTCACGAATTTGCGTGTCAT

Reverse: GCTTCGGCAGCACATATACTAAAAT

18s:

Forward: TAGAGGGACAAGTGGCGTTC

Reverse: CGCTGAGCCAGTCAGTGT

### Knockdown of lncRNAA02Rik by siRNA and shRNA

LncRNAA02Rik-specific siRNA (siA02Rik) and shRNA (shA02Rik) were commercially manufactured by GenePharma (Shanghai, China) as well as IBSBIO Tech. According to the manufacturer’s protocol, the cells were transfected with siA02Rik/shA02Rik (1 µg/mL) and X-tremeGENE Transfection Reagent (Roche, Penzberg, Germany) with 300 μl Serum-free Medium for 5 min. Subsequently, the two reagents were mixed and combined for 18 min at room temperature, after which the mixture was put into cells for 6 h at 37 °C. Thereafter, cardiomyocytes were maintained in the culture medium for 48 h until subsequent experiments.

The sequences of siA02Rik-1 were Forward: CAGAGACUGUCAAGAGUCAGA, Reverse: UGACUCUUGACAGUCUCUGUA; siA02Rik-2 sequences were Forward: GAAUGAUCACUCUGUUAAAUU, Reverse: UUUAACAGAGUGAUCAUUCCA; siA02Rik-3 sequences were Forward: GGACUGUGCUCAAGGCACAGA, Reverse: UGUGCCUUGAGCACAGUCCUG; the sequences of siRNA negative control (siN.C) were Forward: UUCUCCCAACGUGUCACGUTT, Reverse: ACGUGACACGUUCGGAGAATT; shA02Rik sequences were GGACTGTGCTCAAGGCACAGA; the sequences of shRNA negative control (shN.C) were CCTAAGGTTAAGTCGCCCTCG.

Cells were separated into four groups: (1) Control group; (2) Ang-II group: cells were treated with 100 nmol/L Ang-II for 24 h; (3) Ang-II + siA02Rik/Ang-II + shA02Rik group: cells were transfected with siA02Rik/shA02Rik for 24 h then removed it and treated with 100 nmol/L Ang-II for 24 h; (4) N.C group: cells were transfected with siN.C/shN.C for 24 h then removed it and treated with 100 nmol/L Ang-II for 24 h.

### Overexpression of lncRNAA02Rik by plasmid

LncRNAA02Rik cDNA was combined with the pcDNA3.1 vector. An empty vector was regarded as the negative control. Similar to siRNA transfection, plasmid vectors were transfected into cells at a 2.5 mg/L concentration.

Cells were separated into four groups: (1) Control group; (2) lncRANAA02Rik group: lncRNAA02Rik plasmid was transfected into cells for 24 h; (3) Vector group: pcDNA3.1 empty vector was transfected into cells for 24 h; (4) Ang-II group.

### miRNA transfection

The mimics and inhibitors of miR-135a-5p were synthesized by GenePharma and the protocol was the same as that for siRNA transfection.

The sequences of miR-135a-5p mimics were Forward: UAUGGCUUUUUAUUCCUAUGUGA, Reverse: ACAUAGGAAUAAAAAGCCAUAUU; miR-135a-5p inhibitor (AMO-miR-135a-5p) sequences were UCACAUAGGAAUAAAAAGCCAUA; negative control (N.C) sequences were Forward: UUCUCCGAACGUGUCACGUTT, Reverse: ACGUGACACGUUCGGAGAATT; negative control inhibitor (AMO-N.C) sequences were CAGUACUUUUGUGUAGUACAA.

Cells were separated into six groups: (1) Control group; (2) Ang-II group; (3) Ang-II + miR-135a group: cells were treated with Ang-II for 24 h then removed and transfected with miR-135a-5p mimics for 24 h; (4) Ang-II + miR-135a+AMO-135a group: cells were treated with Ang-II for 24 h then removed it and transfected with miR-135a-5p mimics and AMO-miR-135a-5p for 24 h; (5) Ang-II + N.C group: cells were treated with Ang-II for 24 h then removed it and transfected with the negative control for 24 h; (6) Ang-II + AMO-N.C group: cells were treated with Ang-II for 24 h then removed it and transfected with the negative control inhibitor for 24 h.

### Knockdown of TCF7 by siRNA

siRNA targeting TCF7 (siTCF7), and non-targeting control were synthesized by GenePharma. The transfection of siTCF7 was similar to that of siA02Rik/shA02Rik.

The sequences of siTCF7 were Forward: GGAAGAGAGGACAAGGAAUTT, Reverse: AUUCCUUGUCCUCUCUUCCTT.

The sequences of negative control (N.C) were Forward: UGGAGCAAGUUUGGCAGGAGCUAUU, Reverse: AAUAGCUCCUGCCAAACUUGCUCCA.

Cells were separated into four groups: (1) Control group; (2) Ang-II group; (3) Ang-II + siTCF7 group: cells were treated with Ang-II for 24 h then removed it and transfected with siTCF7 for 24 h; (4) Ang-II + N.C group: cells were treated with Ang-II for 24 h then removed it and transfected with the negative control for 24 h.

### Luciferase reporter assays

A lncRNAA02Rik fragment with miR-135a binding sites and TCF7 3′-UTRs was magnified by PCR. Briefly, HEK293 cells were incubated in 48-well culture plates. Lipofectamine 2000 (Invitrogen, Waltham, MA, USA) was used to transfect miR-135a-5p mimics at 10 pmol and luciferase reporter vectors at 40 ng/well. A dual-luciferase reporter assay kit (Promega) was used to detect firefly and renilla luciferase activity. Luciferase activity was normalized using firefly luciferase against renilla luciferase.

### Adeno-associated virus-9-shA02Rik (AAV9-shA02Rik) infection in vivo

AAV9-shA02Rik and AAV9-shN.C were injected into mice via the tail vein (units: 1.2 × 10^12^ vg/mL, 100 μl, IBSBIO Tech). Sham group mice were treated similarly to TAC group mice but with 100 μl saline.

Mice were separated into four groups: (1) Sham group; (2) TAC + N.C group: saline was injected into mice on the second day after TAC for 3 weeks; (3) TAC + shA02Rik group: AAV9-shA02Rik was injected into mice on the second day after TAC for 3 weeks; (4) TAC + shN.C group: AAV9-shN.C was injected into mice on the second day after TAC for 3 weeks.

### Statistical analysis

All statistical analysis was performed using SPSSv19.0 (SPSS Inc, Chicago, IL, USA), and data were presented as mean ± SD. The Student’s *t*-test was used to compare differences between two groups. A one-way ANOVA was used to compare differences among groups. If the ANOVA was significant, SNK-q was used to evaluate the statistical significance of differences between the two groups. *P* < 0.05 was considered to be statistically significant.

## Supplementary information

Supplementary Figure 1

## Data Availability

The datasets used and analyzed during the current study are available from the corresponding author on reasonable request.
