# Supplementary figures and images for: LncRNA4930473A02Rik promotes cardiac hypertrophy by regulating TCF7 via sponging miR-135a in mice
Source: Cell Death Discov. 2021 Dec 7;7:378. doi: 10.1038/s41420-021-00775-8 (PMC8651675; doi:10.1038/s41420-021-00775-8)

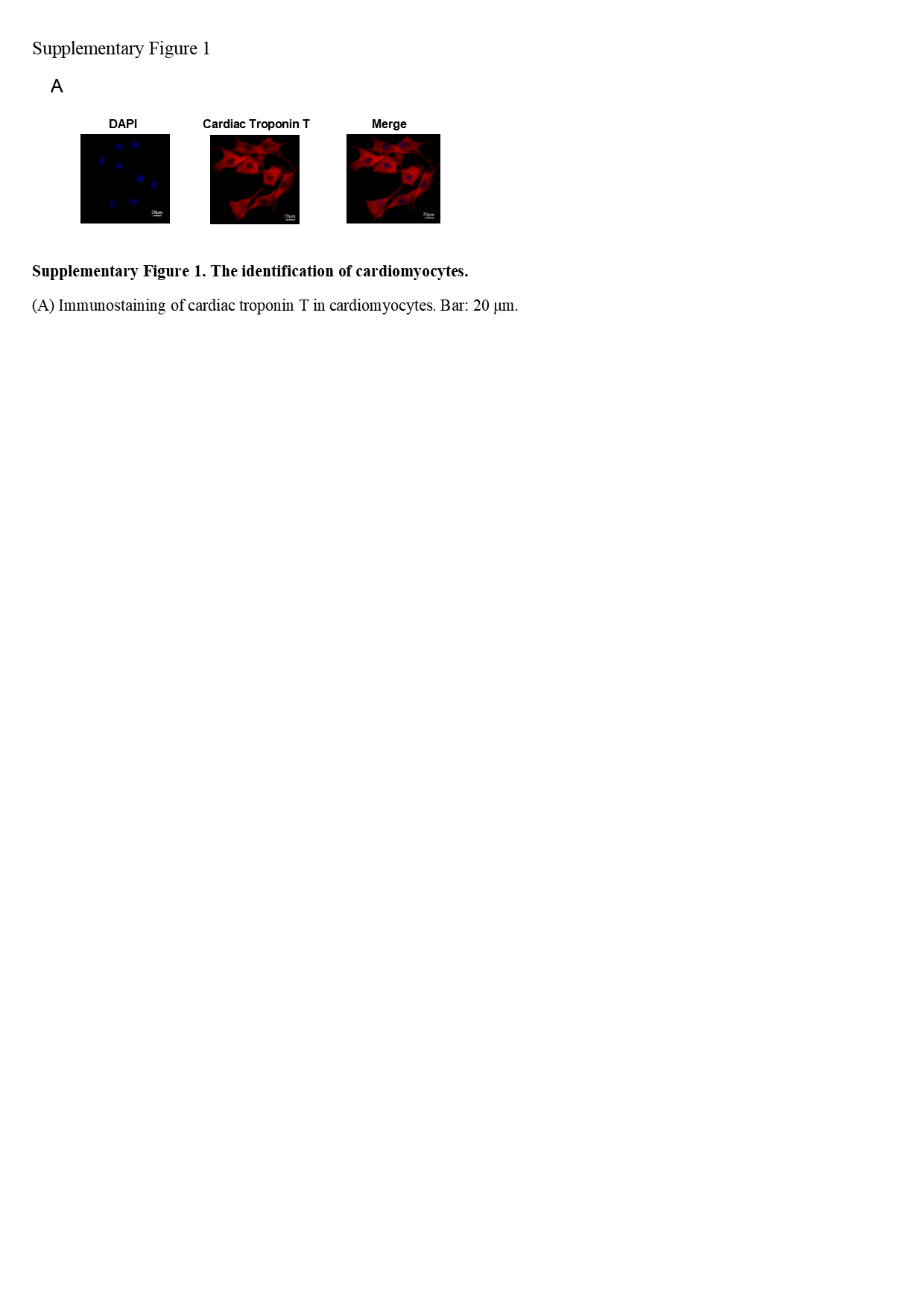

Supplement: Supplementary file 1 — Supplementary Figure 1 [file 41420_2021_775_MOESM1_ESM.tif]
